# Safer prescription of drugs: impact of the PREFASEG system to aid clinical decision-making in primary care in Catalonia

**DOI:** 10.1186/s12911-021-01710-8

**Published:** 2021-12-15

**Authors:** M. Àngels Pons-Mesquida, Míriam Oms-Arias, Eduard Diogène-Fadini, Albert Figueras

**Affiliations:** 1 Unitat de Coordinació i Estratègia del Medicament (UCEM), Institut Català de la Salut, Barcelona, Spain; 2 Servei de Farmacologia Clínica, Institut Català de la Salut, Hospital Universitari Vall d’Hebron, Barcelona, Spain; 3 Departament de Farmacologia, Terapèutica i Toxicologia, Universitat Autònoma de Barcelona, Barcelona, Spain

**Keywords:** Clinical decision support system, Primary care, Clinical safety, Electronic prescription, Pharmacovigilance, Medicines use

## Abstract

**Background:**

In 2008, the Institut Català de la Salut (ICS, Catalan Health Institute) implemented a prescription decision support system in its electronic clinical workstation (ECW), which automatically generates online alerts for general practitioners when a possible medication-related problem (MRP) is detected. This tool is known as PREFASEG, and at the time of beginning a new treatment, it automatically assesses the suitability of the treatment for the individual patient. This analysis is based on ongoing treatments, demographic characteristics, existing pathologies, and patient biochemical variables. As a result of the assessment, therapeutic recommendations are provided. The objective of this study is to present the PREFASEG tool, analyse the main alerts that it generates, and determine the degree of alert acceptance.

**Methods:**

A cross-sectional descriptive study was carried out to analyse the generation of MRP-related alerts detected by PREFASEG during 2016, 2017, and 2018 in primary care (PC) in Catalonia. The number of MRP alerts generated, the drugs involved, and the acceptance/rejection of the alerts were analysed. An alert was considered "accepted" when the medication that generated the alert was not prescribed, thereby following the recommendation given by the tool. The MRP alerts studied were therapeutic duplications, safety alerts issued by the Spanish Medicines Agency, and drugs not recommended for use in geriatrics. The prescriptions issued by 6411 ICS PC physicians who use the ECW and provide their services to 5.8 million Catalans through 288 PC teams were analysed.

**Results:**

During the 3 years examined, 67.2 million new prescriptions were analysed, for which PREFASEG generated 4,379,866 alerts (1 for every 15 new treatments). A total of 1,222,159 alerts (28%) were accepted. Pharmacological interactions and therapeutic duplications were the most detected alerts, representing 40 and 30% of the total alerts, respectively. The main pharmacological groups involved in the safety alerts were nonsteroidal anti-inflammatory drugs and renin-angiotensin system inhibitors.

**Conclusions:**

During the period analysed, 28% of the prescriptions wherein a toxicity-related PREFASEG alert was generated led to treatment modification, thereby helping to prevent the generation of potential safety MRPs. However, the tool should be further improved to increase alert acceptance and thereby improve patient safety.

**Supplementary Information:**

The online version contains supplementary material available at 10.1186/s12911-021-01710-8.

## Background

According to a European Commission report, 3–10% of hospital admissions between 2012 and 2014 were caused by adverse drug events (ADEs), totalling 2.5–8.4 million cases annually. In addition, approximately 2.1–6.5% of hospitalised patients experienced an ADE, corresponding to 1.8–5.5 million annually [1]. Thus, since the late 1990s, patient safety has become a priority of health systems [2, 3], and several initiatives have identified the need for a new culture of safety in the health and policy environment [4–6]. To achieve this, and according to the definition of clinical safety, it is essential to define actions to avoid, prevent, and improve adverse effects or injuries from healthcare processes where possible, since it should be acknowledged that some adverse events are inherent in treatment, and cannot always be avoided or minimised.

In this context, the 1999 technical report ‘To err is human’ by the Institute of Medicine (OIM) highlighted the need to develop new information and communications technologies to reduce medical errors [2], and, beyond this, prescriptions which could increase the risk of developing adverse effects. Subsequent reports later affirmed that the electronic record of healthcare activity that is typical of an electronic health record (EHR), together with the integration of clinical decision support systems (CDSSs) into these EHRs, should contribute to guaranteeing quality in the healthcare system [7, 8] by helping to reduce preventable adverse effects.

In the scientific literature we find different definitions of a CDSS [9, 10]. According to Kawamoto et al. [11], a CDSS can be considered any electronic system designed to help clinical decision making, which takes into account the characteristics of the patient to generate a specific evaluation and provide a recommendation to be evaluated by the practicing clinician. The design and functionalities of these CDSSs can be very varied. Some authors consider that CDSSs aimed at the initial prescription phase may have the greatest impact on improving patient safety [12], while others discuss the fact that integration of a CDSS into the HER renders it possible to provide patient histories along with interactive signals that alert professionals to situations of risk for the patient [13]. As a result, the prescription process can be improved and the clinical safety of patients enhanced [12, 14, 15].

CDSSs have been found to bring multiple benefits to patient care [16–19], wherein it has been reported that they contribute to improving the dosage and selection of drugs, while also encouraging patients to take part in preventive activities, improving test results, decreasing morbidity, and improving the quality of care [20]. In contrast, the main risk of CDSSs is the alert fatigue experienced by physicians who are faced with a multitude of prompts and reminders on-screen, which can lead to important alerts being ignored [21, 22].

The Catalan Health Institute (ICS) is a public entity that provides health services to 80% of the population of Catalonia. In 2008, in line with promoting the clinical safety of the patient, it designed and integrated a CDSS into its primary care electronic clinical workstation (ECW) that made it possible to detect certain medication-related problems (MRPs) online. This CDSS, which is known as PREFASEG (PREscripción FArmacéutica SEGura, i.e., safe pharmaceutical prescription), is a computer tool that acts interactively to alert clinicians to any potential drug use-related problems during the process of deciding the most appropriate treatment for their patient.

**Fig. 1:**
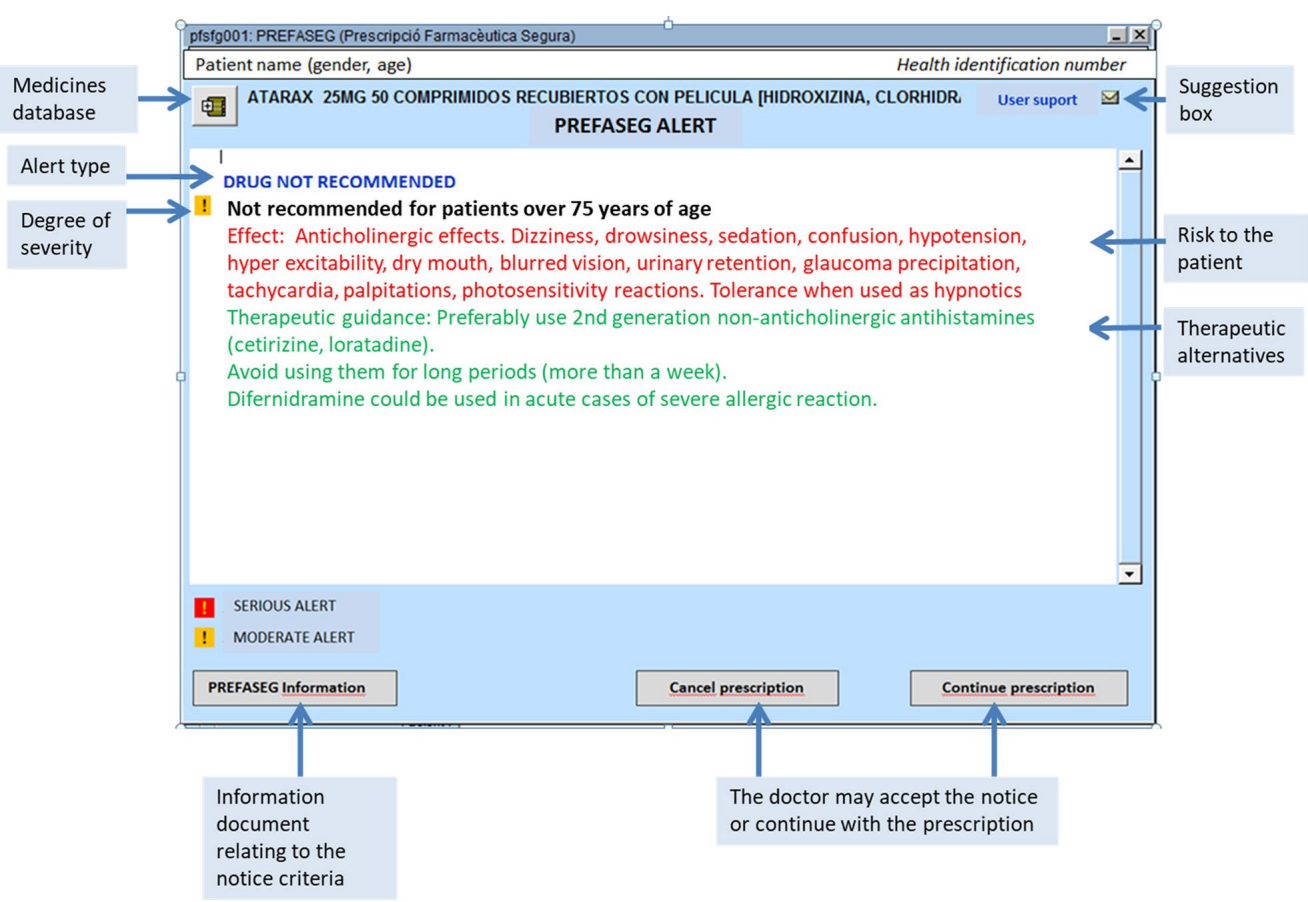
Example PREFASEG screen showing the various informative elements presented by the tool (screenshot obtained from the PREFASEG ECW and translated into English)

To understand the means by which PREFASEG functions, we consider the prescription of a new drug to a specific patient. At the point at which the prescription is requested by the clinician, PREFASEG is activated and performs an assessment of the prescription to verify that it is safe for the patient, and that it does not pose a potential risk to their health. This evaluation is carried out based on the different MRPs detected by the tool, which include: (1) Drug interactions; (2) Therapeutic duplications; (3) Drugs advised against for use in geriatrics; (4) Contraindications with a safety alert published by the Spanish Agency for Medicines and Health Products (AEMPS, Agencia Española del Medicamentos y Productos Sanitarios); (5) Contraindications due to health problems and/or clinical variables; (6) Drugs that are known to be teratogens during pregnancy; (7) Anticholinergic drug combinations; (8) Patient history of hypersensitivity or suspected hypersensitivity reactions (suspected, not confirmed); and (9) Adverse drug events. To carry out this evaluation, a number of factors are taken into account, such as any active prescriptions that the patient already has on their record, other medical diagnoses or active health problems, the presence of any clinical variables with altered values, and the age and/or sex of the patient. In the event of a safety alert being generated following the above evaluation, the corresponding warnings are shown to the clinician (e.g., the risk to the patient and any therapeutic alternatives) so that he can decide whether to continue with the prescription or change the medication. These safety alerts are displayed in a simple manner on a single screen to permit their rapid consultation and understanding, as shown in Fig. 1, which presents an example relating to the prescription of a product that is not recommended for patients over 75 years of age. The information displayed includes the severity icon (two degrees, moderate or severe), the drug or active ingredient causing the alert, the cause of conflict (i.e., medication or active ingredient conflict, patient age, or pre-existing health problem), the risk to the patient, and any therapeutic alternatives. Each MRP alert is classified as either high (red indicator) or medium–low (orange indicator) clinical relevance, according to previously described recommendations [23].

**Fig. 2:**
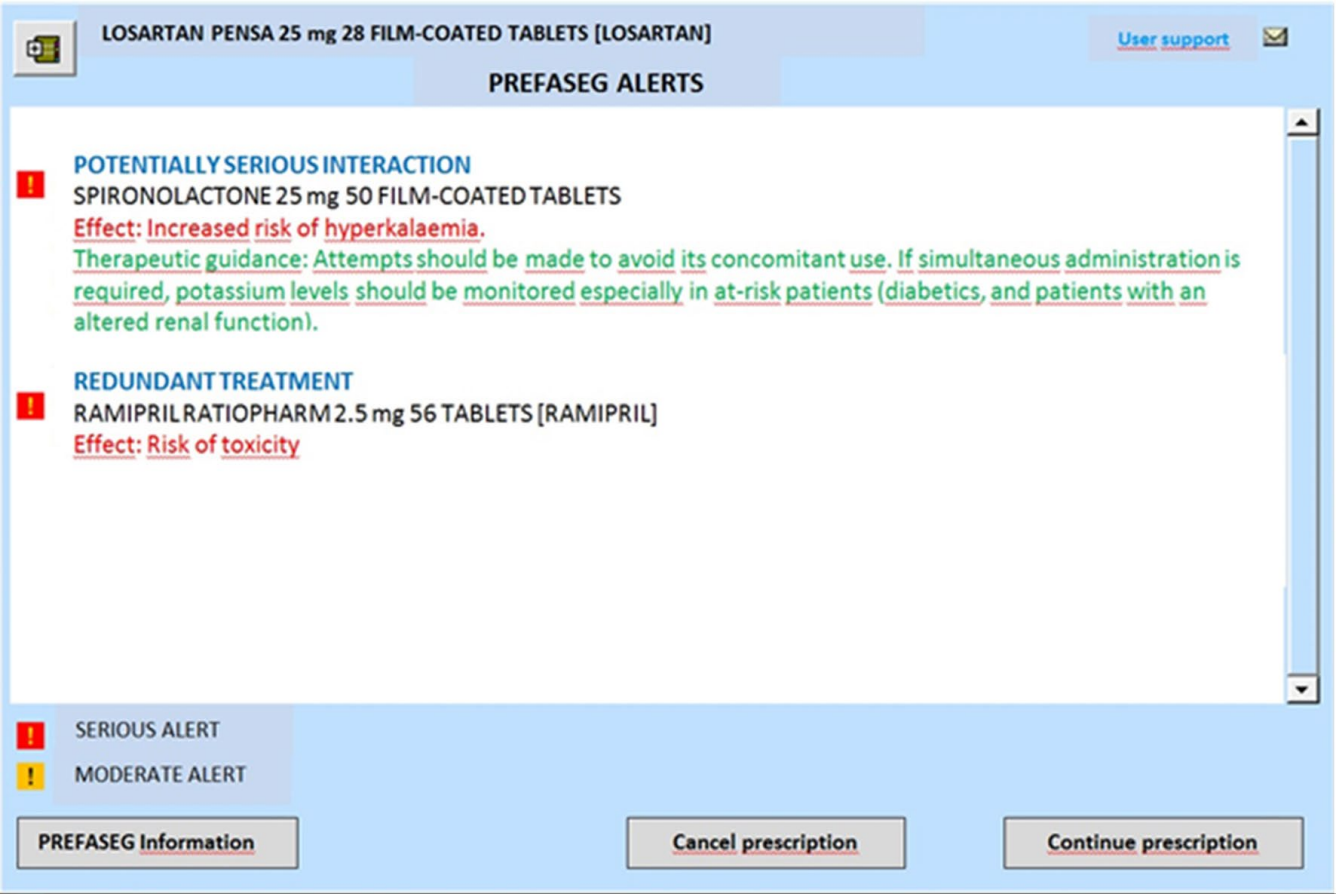
Example screen of the PREFASEG system with various included safety alerts and therapeutic recommendations (screenshot obtained from the PREFASEG ECW and translated into English)

It is also possible that more than one alert is generated by the tool, and in such a case, the clinician is informed of all warnings associated with the different potential safety issues, as can be seen in Fig. 2.

Each alert generated by PREFASEG is recorded as "audit data” along with the information related to whether it has been accepted or whether prescription of the product was continued. The evaluation of these data allows us to determine if certain types of alerts are accepted to a greater degree than others, thereby improving the clinical content definitions to adapt the tool to the healthcare reality. PREFASEG consists of a calculation core in the Oracle PL/SQL, in which the calculations that access the tables of clinical contents have been optimised, and in which there is a minimum visual interface for the communication of safety alerts. This interface was developed using Developer Forms, which is the same technology employed to produce the ECW. As a result, the look and feel of the alert screens are comparable to those of the original ECW, and so maximum integration is achieved.

The contents of the MRP alerts are defined and maintained as described by a multidisciplinary group of expert professionals from the ICS (i.e., primary care (PC) physicians, pharmacists, and clinical pharmacologists) according to previous literature [9, 24]. The MRP alerts are reviewed and updated each year according to the available scientific evidence, and as a result, the clinical content undergoes some changes from 1 year to the next. For example, references to the more current STOPP/START and Beers criteria were included in updates for drugs advised against for use in geriatrics. Importantly, the clinical content can be revised at any time, and are updated from a specific maintenance platform known as 'Know How.'

The purpose of this study is therefore to describe the principal characteristics of the PREFASEG tool, the main safety alerts generated by PREFASEG in the Catalan PC system, the degree of acceptance of these alerts by physicians, and the main pharmacological groups implicated in the alerts. Furthermore, three of the most frequent alerts are also described in greater detail.

## Methods

A descriptive, cross-sectional study was designed, which began in January 2016 and continued until December 2018. This study was developed within the scope of the PC system of the ICS, which is the main entity that provides health services in Catalonia, and which covers a population of 5.8 million inhabitants of the different Catalan territories through a network of 288 PC teams and 8 hospitals. The ICS is a public company with a total of 42,374 professionals who provide services to 80% of the population of Catalonia.

### Study sample

The sample studied consisted of all prescriptions issued by the 6411 ICS PC physicians who used the EHR during the study period.

### Development of PREFASEG

In general terms, to provide the PREFASEG with clinical and pharmacological content, the following methodology was followed during its development:

1. Bibliographic search. Initially a bibliographic search was carried out in the PubMed database for the different MRPs addressed by PREFASEG, wherein national and international articles that were considered to be the most relevant and best adapted to our healthcare environment were reviewed. The safety alerts included in the ICS Clinical Practice Guidelines [25] were also considered.
2. Consensus with a group of experts. Following a literature review, the pharmacological groups to be included were selected and the messages to be presented to the prescribing physicians were defined. Each MRP alert was classified as high (red) or medium-low (orange) clinical relevance, as mentioned above [23]. The red alerts reflected situations of absolute contraindications, while the orange alerts were considered precautionary.
3. Adaptation of the clinical content to the table formats necessary for the PREFASEG computer program. The clinical content was transferred into a computer-readable language from various configuration tables presented in Excel, and for this purpose, it was necessary to code the active ingredients according to the Anatomical Therapeutic Chemical (ATC) classification. Similarly, the various health issues were coded according to the International Classification of Diseases ICD-10 system. To produce the clinical contents, ATC groups or groups of health problems were built. Each alert type was then defined and configured using a combination of various attributes, as outlined in Table 1.

**Table 1:** Combinable attributes in the configuration of each PREFASEG notice

Type of alert	Combinable attributes in the PREFASEG message configuration
Interactions	ATC drug groups
Duplicate therapies	ATC drug groups
AEMPS safety alerts	Age
	ATC drug groups
	Grouping based on health problems
	Dose of the active ingredient that generates the warning
Advised against for use in geriatrics	Age
Contraindications due to health issues	ATC drug groups
Contraindications due to clinical variables	Grouping based on health problems
Teratogens in pregnancy	Labelling of clinical variables (e.g., glomerular filtration and potassium levels)
Combinations of anticholinergic drugs	ATC drug groups
Suspicions of hypersensitivity	ATC drug groups
Adverse drug reactions	ATC drug groups

### Variables and indicators

The main variable of the study was the number of MRP alerts generated by PREFASEG. Another of the variables studied was the number of accepted alerts. An alert was considered "accepted" when the medicine that generated the safety alert was not prescribed.

Some PREFASEG alerts are associated with recommendations for clinical follow-ups or dose reductions. Therefore, following these recommendations does not entail the withdrawal of the treatment that has generated the alert. Consequently, these alerts are not considered “accepted” alerts.

### Description of the MRPs included in PREFASEG

The various MRPs that are defined in the PREFASEG system were outlined previously in the introduction (see also Table 1). Further details regarding these MRPs can be found in Additional file 1.

The global MRP alerts generated and accepted by PREFASEG were analysed. More specifically, the safety MRPs related to therapeutic duplications, medicines not recommended for use in geriatrics, and safety alerts from the AEMPS were examined in greater detail.

The contents of the MRP alerts were defined and maintained as described by a multidisciplinary group of expert professionals from the ICS according to previous literature [9, 24]. The MRP alerts were reviewed and updated each year according to the available scientific evidence, and as a result, the clinical content underwent some changes from 1 year to the next. Each MRP alert was classified as either high or medium–low clinical relevance, as described above [23].

The MRP alerts corresponding to “therapeutic duplications” detected patients with a non-beneficial prescription of two or more medicines based on the same active ingredient (alone or in combination) and/or with the same pharmacological action (further details can be found in Additional file 1). Duplications of more than 60 different pharmacological groups commonly used in PC were addressed. In each group, “clinically relevant duplications” and “dose adjustments duplications” (combinations sought with a therapeutic objective) were clearly differentiated. Depending on their relevance, alerts marked with different colours were generated, as indicated above.

During the study period, MRP alerts associated with “AEMPS safety alerts” reported contraindications for the “Triple Whammy,” COXIBS, diclofenac, aceclofenac, cilostazol, ivabradine, agomelatine, escitalopram, citalopram, trimetazidine, raloxifene/bazedoxifene, strontium ranelate, aliskiren, and canagliflozin (further details can be found in Additional file 1). These alerts were considered to be highly relevant because they were absolute contraindications, in addition to having a specific safety alert originating from the AEMPS, and so they were indicated in red.

The MRP alerts corresponding to “medicines not recommended for use in geriatrics” detected patients ≥ 75 years of age who had been prescribed inappropriate medication that posed a more unfavourable risk–benefit profile due to their age (see Additional file 1). The selection of medications considered inappropriate for this age group was based on the Beers (2015) [26], EU-PIM (European Consensus) [27], STOPP/START [28], and PRISCUS [29] criteria (further details can be found in Additional file 1). These alerts were displayed on-screen as alerts of medium–low relevance (i.e., orange colour) since the literature indicates that they should be administered with caution.

### Data collection and analysis

The analysed data were obtained from the ECW that stores the active prescriptions of all patients; however, data from specific patients were not analysed. The study was restricted to drugs prescribed and financed by the National Health System for use in the PC setting.

In January 2016, information began to be extracted regarding the different types of MRP alerts generated by PREFASEG, which were internally identified in the patient's EHR. Thus, the number of advisories for each MRP generated, the medicines involved in each alert, and the acceptance or rejection of the alert were recorded. Each month, the alerts generated by the system and accepted by the clinicians were accumulated in a computer repository. The data set was analysed annually through computerised extractions from the ECW databases. The alert traceability was stored and organised on computer servers according to the organisational structure of the ICS, i.e., with differentiation between the health territories in which the institution is organised.

A descriptive analysis was carried out of the generated and accepted alerts of the different MRPs from January 2016 to December 2018. Initially, the analysis was carried out on an annual basis because the clinical contents changed annually. These content changes occurred for a number of reasons, including the inclusion of new marketed drugs, modifications in the definitions of existing MRP alerts to render them more specific, and the inclusion of additional pharmacological groups. Despite these content changes, the data were accumulated, and a global analysis of the alerts generated and accepted during the 3-year study period was also carried out.

## Results

### General analysis of the MRP alerts generated by PREFASEG

During the period of study, 22.5, 22.3, and 22.4 million new prescriptions were issued in the ICS PC system in 2016, 2017, and 2018, respectively, while the number of alerts generated by PREFASEG were 1.17 million in 2016, 1.43 million in 2017, and 1.77 million in 2018. Thus, the percentage of MRP alerts generated by the tool with respect to the number of new prescriptions issued were 5% in 2016, 6% in 2017, and 8% in 2018.

The global acceptance of these alerts varied throughout the 3 years studied, ranging from 31% (362,732) in 2016 to 26% (457,976) in 2018 (see Table 2), which corresponds to 69–74% of the MRP alerts generated by PREFASEG during the years of study. Analysis of the accumulated number of alerts issued over the 3-year study period (i.e., 4.38 million alerts) gave a 28% degree of acceptance (i.e., 1.22 million accepted alerts).

**Table 2:** MRP alerts generated and accepted by PREFASEG between January 2016 and December 2018

PREFASEG	2016			2017			2018			Sum 2016–2018		
Type of MRP alert	Alerts generated	Alerts accepted	% Accepted alerts	Alerts generated	Alerts accepted	% Accepted alerts	Alerts generated	Alerts accepted	% Accepted alerts	Alerts generated	Alerts accepted	% Accepted alerts
Interactions	439,507	118,485	27	550,692	138,947	25	701,687	166,452	24	1,691,886	423,884	25
Duplicate therapies	426,506	141,371	33	463,418	136,097	29	546,797	148,943	27	1,436,721	426,411	30
Advised due to age (> 75 years)	108,974	32,345	30	144,807	41,740	29	188,139	50,299	27	441,920	124,384	28
AEMPS safety alerts	59,146	18,301	31	86,308	21,958	25	84,158	18,720	22	229,612	58,979	26
Contraindications due to health issues	35,164	13,771	39	67,452	21,330	32	105,749	28,390	27	208,365	63,491	30
Teratogens in pregnancy	11,721	4200	36	10,938	3830	35	11,404	3709	33	34,063	11,739	34
Combinations of anticholinergic drugs	1077	423	39	2619	863	33	3171	877	28	6867	2163	31
Suspicions of hypersensitivity	76,883	28,952	38	84,099	29,759	35	93,852	30,568	33	254,834	89,279	35
Adverse drug reactions	15,397	4884	32	23,712	6927	29	36,489	10,018	27	75,598	21,829	29
Totals	1,174,375	362,732	31	1,434,045	401,451	28	1,771,446	457,976	26	4,379,866	1,222,159	28

When analysing the alerts generated from the different MRPs throughout the study period (2016–2018), it was observed that those related to drug interactions, therapeutic duplications, and drugs advised against for use in geriatrics were the most common. Taking the data collected over the 3 years, 39% (1,691,886) of the 4,379,866 million alerts generated were for drug interactions, 33% (1,436,721) were for therapeutic duplications, and 10% (441,920) were for the use of drugs advised against in geriatrics. Thus, these three types of MRP alerts accounted for more than three-quarters of the PREFASEG alerts (3,570,527; 82%). Of these, 27% were accepted and 73% were ignored. In addition, of the 34,063 alerts related to teratogens in pregnancy, 22,324 (66%) were ignored.

The types of alerts with the highest percentage of acceptance were those related to a history of suspected (unconfirmed) drug hypersensitivity, with 35% (89,279) of these alerts being accepted over the 3 years studied. Detailed analyses indicated that four non-steroidal anti-inflammatory drugs (NSAIDs, i.e., ibuprofen, naproxen, desketoprofen, and diclofenac) represented 45% (113,936) of the suspected hypersensitivity reactions reported by PREFASEG, with ibuprofen generating the highest number of alerts (61,026). In addition, during the study period, the number of suspected hypersensitivity reaction alerts for β-lactam antibiotics alone or in combination fell into the second largest group, with 39,622 alerts and an acceptance level of 63% (25,153).

In contrast, the alerts related to potential teratogenic compounds during pregnancy had a lower degree of acceptance (i.e., 34%, 11,739). It was observed that the active ingredients that generated the most alerts were ibuprofen (10,864) and acetylsalicylic acid (4336) out of a total of 34,063.

Overall, the alerts with the lowest degree of acceptance were those attributed to interactions between treatments, with 25% (423,884) of a total of 1,691,886 being accepted. More specifically, the interactions of NSAIDs with acetylsalicylic acid generated the greatest number of alerts, reaching 220,507 alerts with an acceptance level of 18% (40,227). Table 3 outlines the 10 main interactions at the active ingredient level, which represent 30% (506,082) of all alerts of this type.

**Table 3:** Top 10 alerts related to drug interactions between January 2016 and December 2018

Original active ingredient	Conflicting active ingredient	Alerts generated	Acceptance (%)
Ibuprofen	Acetylsalicylic acid	97,847	19
Amlodipine	Simvastatin	85,358	24
Naproxen	Acetylsalicylic acid	64,913	16
Simvastatin	Amlodipine	59,453	26
Dexketoprofen	Acetylsalicylic acid	32,455	16
Acenocoumarol	Simvastatin	31,349	29
Simvastatin	Acenocoumarol	31,349	26
Tramadol and paracetamol	Citalopram	27,479	24
Tramadol and paracetamol	Sertraline	26,872	24
Diclofenac	Acetylsalicylic acid	25,292	24
Enoxaparin	Acetylsalicylic acid	23,715	16

### Analysis of the PREFASEG alerts related to therapeutic duplication

**Fig. 3:**
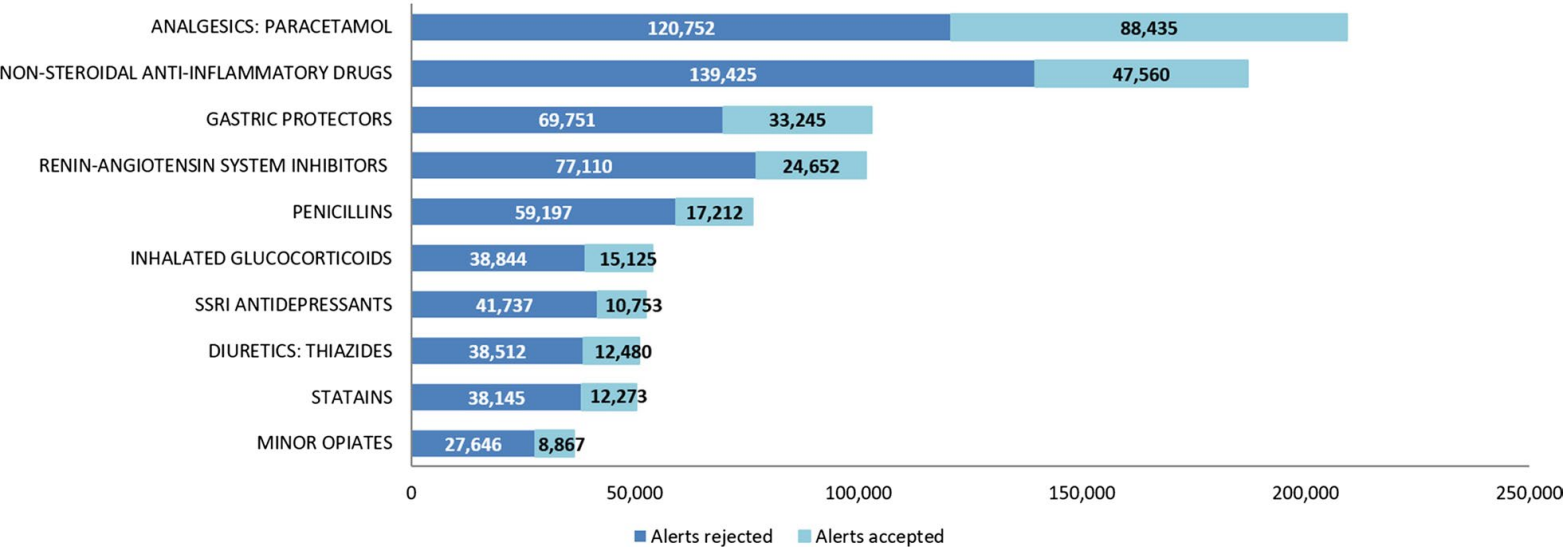
Degrees of detection and acceptance of the main types of duplication from January 2016–2018

In the 3 years studied, the four groups of duplications that generated the most alerts were the NSAIDs, paracetamol-type analgesics, renin-angiotensin system (RAS) inhibitors and gastric protectors (see Fig. 3). Out of a total of 65 groups, these 4 duplication groups represented 42% (600,930) of the total alerts generated (1,436,721). Duplications related to analgesics and gastric protectors had the highest levels of acceptance during the study period, reaching 42% (88,435) and 32% (33,245), respectively. In contrast, duplications related to the SSRI antidepressants and the RAS inhibitor antihypertensives had the lowest degrees of acceptance, i.e., 20% (10,753) and 21% (20,940), respectively.

Furthermore, the pairs of active ingredients with the greatest numbers of alerts generated for duplications during the 3 years studied were paracetamol–paracetamol, paracetamol–paracetamol with tramadol, omeprazole–omeprazole, ibuprofen–ibuprofen, and metamizole–metamizole. These 5 pairs represented 22% (317,390) of the duplications, of which 144,450 (46%) were accepted.

Moreover, the duplications related to antibiotics such as amoxicillin generated a considerable number of alerts, i.e., 76,409 over the 3 years studied, and their acceptance rate was relatively low at 23%. Upon the analysis of other groups of potentially dangerous duplications, such as those related to oral anticoagulants, it was observed that PREFASEG generated 14,903 alerts of duplications for this group, of which 42% (6314) were accepted, and the prescriptions were not continued.

### Analysis of PREFASEG alerts related to AEMPS safety alerts

It was found that the number of alerts related to AEMPS safety alerts increased throughout the study period, i.e., from 59,146 in 2016 to 84,158 in 2018. This was accompanied by a reduction in the degree of acceptance of these alerts from 31% (18,301) to 22% (18,720), respectively.

Analysing the details of these AEMPS alerts (see Table 4), it was apparent that the Triple Whammy, which considered the concomitant therapy of NSAIDs, diuretics, and RAS inhibitors, generated the highest degree of alerts, representing 85% (195,987) of the total alerts. The degree of acceptance of this type of alert varied, ranging from 30% (13,867) in 2016 to 22% (15,988) in 2018, thereby indicating a decrease in acceptance over this 3-year period. Reviewing the main anti-inflammatory drugs that generated the Triple Whammy alerts, it was observed that in 65% of the cases, the NSAIDs involved were ibuprofen and naproxen.

**Table 4:** Degree of detection and acceptance of the AEMPS safety alerts in 2016, 2017, and 2018

	2016			2017			2018		
AEMPS alert	Alerts	Alerts accepted	% Accepted	Alerts	Alerts accepted	% Accepted	Alerts	Alerts accepted	% Accepted
"TRIPLE WHAMMY"*	46,020	13,867	30	76,107	18,992	25	73,860	15,988	22
DICLOFENAC	7288	2588	36	5734	1774	31	5137	1511	29
COXIBS	3131	888	28	2535	616	24	2986	644	22
ACECLOFENAC	805	204	25	665	127	19	487	101	21
ESCITALOPRAM	645	204	32	270	95	35	305	72	24
CITALOPRAM	495	189	38	217	67	31	302	104	34
CILOSTAZOL	193	70	36	181	66	36	289	65	22
AGOMELATINE	133	54	41	175	54	31	249	72	29
IVABRADINE	130	51	39	153	56	37	200	48	24
CANAGLIFLOZIN	–	–	–	128	42	33	177	40	23
TRIMETAZIDINE	115	62	54	82	36	44	111	47	42
RALOXIFENE and BAZEDOXIFENE	106	64	60	23	11	48	30	20	67
STRONTIUM RENALATE	53	39	74	21	11	52	22	5	23
ALISKIREN	32	21	66	17	11	65	3	3	100
Total	59,146	18,301	31	86,308	21,958	25	84,158	18,720	22

*Triple Whammy: NSAIDs + RAS inhibitors + diuretics

As indicated in Table 4, the number of alerts generated by diclofenac decreased over time. More specifically, in 2016, diclofenac generated 7288 alerts, while by 2018 this number had reduced to 5137. However, the degree of acceptance also decreased from year to year, dropping from 36% (2588) in 2016 to 29% (1511) in 2018.

The alerts with the highest degree of acceptance were those related to strontium ranelate, raloxifene/bazedoxifene, and aliskiren; however the number of such alerts was low since these are drugs that are being gradually withdrawn from the market, or tend to be unused in daily practice.

### Analysis of the PREFASEG alerts related to medicines not recommended for use in geriatrics between January 2016 and December 2018

The alerts generated by PREFASEG that were related to drugs advised against for use in geriatrics increased throughout the study period, i.e., from 108,974 in 2016 to 188,139 in 2018. However, a reduction in the degree of acceptance from 30% (32,345) to 27% (50,299), respectively, was also observed.

The two pharmacological groups that generated the highest number of alerts within this category were the benzodiazepines and the NSAIDs, representing 39% (172,574) and 21% (47,966) of a total of 441,920 alerts in the 3 years studied (see Table 5). More specifically, the alerts related to benzodiazepine usage in geriatrics increased by 55% during the study period, i.e., from 30,662 in 2016 to 68,974 in 2018. Alprazolam represented 44% (65,910) of the alerts generated for benzodiazepines in this group of patients (see Table 6), and overall, the benzodiazepine group showed an acceptance rate of 27% over the 3 years.

**Table 5:** Pharmacological groups not recommended in the elderly that generated PREFASEG alerts from January 2016–December 2018

Not recommended pharmacological groups	Alerts generated	Total accepted	% Accepted
Benzodiazepines, hypnotics, and sedatives	172,574	47,966	27
Anti-inflammatory and anti-rheumatic (NSAIDs, COXIBS)	94,034	20,627	22
Antihypertensives	34,542	10,310	30
Digestive system (otilonium, metoclopramide, glibenclamide, chlorporpamide)	31,496	8945	28
Chronic obstructive pulmonary disease treatments (theophylline)	19,285	4758	25
Central action muscle relaxants (cyclobenzaprine)	18,170	7521	41
Tricyclic antidepressants and Fluoxetine	17,289	5654	33
Peripheral vasodilators (pentoxifilline, nicergoline, nafthydrofuril)	16,958	6621	39
Respiratory system (systemic antihistamines)	14,219	4917	35
Hormone therapy (megestrol)	10,776	2631	24
Urinary antispasmodics (oxybutynin)	7018	2495	36
Antithrombotics (cilostazol)	3270	1100	34
Beta-blockers (sotalol)	1105	392	35
Opioid and anti-migraine pain relievers	868	342	39
Antiparkinsonian drugs	316	105	33

**Table 6:** Top 10 alerts for drugs advised against for use in geriatrics between January 2016 and December 2018

Active ingredient responsible for the alert	Alerts generated	Acceptance (%)
Alprazolam	65,910	24
Dexketoprofen	45,293	24
Doxazosin	33,867	30
Clonazepam	23,887	35
Zolpidem	23,314	25
Pentoxifylline	20,395	31
Metoclopramide	19,098	28
Hydroxyzine	18,328	29
Etoricoxib	15,729	18
Potassium clorazepate	15,468	29

Within the NSAID alerts, it was observed that desketoprofen represented 48% (45,293) of the total alerts in this group, and this group showed one of the lowest levels of acceptance (i.e., 22%).

In general, analysis of the degrees of acceptance in this class of alerts shows a significant level of variation (see Table 5), and the pharmacological groups with the highest degree of acceptance (i.e., where no prescription was issued for the corresponding treatment) were the muscle relaxants (41%, 7521) and the peripheral vasodilators (39%, 6621).

## Discussion

The main finding of this study was that the PREFASEG system appears to adopt the role of a CDSS that assists in preventing potential safety MRPs for patients by generating online alerts when starting a new treatment. During the period studied, it was observed that 28% of the generated security alerts led to a modification of the prescription (i.e., acceptance of the alert). In absolute terms, between 2016 and 2018, a total of 1,222,159 recommendations were accepted globally, which likely led to the avoidance of numerous potential MRPs in patients. Overall, PREFASEG reported a safety MRP in 1 out of every 15 new prescriptions. The degrees of acceptance of the recommendations were relatively high when compared with a similar study into a different online preventive alert system, where the percentages of acceptance ranged from 12 to 14% [30]. However, the variability between studies was considerable; in a 2009 Cochrane review on the effects of online prompts/reminders, an improvement of only 4.2% was reported [18], while other studies described omissions of recommendations in between 49 and 96% of the cases [31]. In general, subsequent systematic reviews [32, 33] concluded that online notification systems had only a small or moderate effect.

Our study presents a number of characteristics that could explain the relatively high acceptance rate of alerts. For example, the observed degree of acceptance could be accounted for by considering that the tool takes into account the success characteristics of CDSSs described by various authors previously [10, 34]. More specifically, it is integrated into the workflow of clinicians, it generates an alert automatically during the parent consultation and in the context of their medical history, and it provides a specific therapeutic recommendation in each case. In addition, PREFASEG produces different types of safety alerts based the clinical situation of each patient and any medication that they may be taking, and it gives alerts related to the safe prescription of medication for various pathologies. To date, few studies have analysed such a diversity of alerts simultaneously [35–37]. It should also be emphasised here that PREFASEG is a tool whose clinical contents are constantly being updated and that has been in use for more than 12 years, during which time it has exhibited a good degree of acceptance by a large number of medical professionals, since it is used by > 6400 PC physicians on a daily basis. Indeed, both ICS pharmacists and PC clinical pharmacologists promote the use of this tool.

Despite the high number of potential MRPs avoided in patients in Catalonia, it should be noted that in the case of safety alerts relating to the use of medicines, it was striking that globally, > 70% of the alerts generated by PREFASEG were ignored, which amounts to 3.16 million over the 3-year study period. However, it must be considered that, on occasions, the recommendations given by PREFASEG did not necessarily imply a change, or the change was not recorded as such. In addition, some recommendations involved a dose reduction or a clinical follow-up of some variable, and therefore the prescription was continued. In such circumstances, the system did not count the recommendation as accepted, even if the dose was lowered or an analysis was requested. If these modifications had been registered, the degree of acceptance would be higher, as the presented acceptance values relate only to cases where the prescription was continued. It should also be considered that not all alerts had the same degree of clinical relevance, with alerts being accompanied by either an orange or a red icon, depending on the importance of the recommendation, as also described in a previous study [23].

Over the course of the 3 years studied, the number of generated alerts increased. This was partly related to the fact that new and updated content was introduced into the PREFASEG system on an annual basis. The existence of a direct relationship between the increased consumption of certain drugs (e.g., the benzodiazepines) was also considered, in addition to the probability that greater numbers of MRP alerts could be generated from such drugs due to their increased use among the population. At the same time, it should be noted that the degree of acceptance of the alerts tended to decrease over time, with practitioners gradually ignoring the recommendations. This decline in acceptance could be partly attributed to alert fatigue; however, it will be necessary to further investigate the reasons behind the rejection of alerts, in addition to separately analysing the high and medium–low relevance alerts, while also considering the cases where the recommendation does not suggest a change of drug. It will also be essential to collect the opinions of the professionals who use the PREFASEG tool. According to various reports, the main reasons for low acceptance by clinicians are the large number of low-relevance alerts they receive and their poor content [21, 22, 38]. To reduce the risk of fatigue, it is therefore necessary to increase the specificity of the alerts, provide clear and concise information, and not impact on the clinician’s workflow.

In relation to the ignored alerts regarding suspicions of a history of hypersensitivity to certain drugs, it is known that general practitioners tend to register cases of hypersensitivity that are reported by patients, despite the fact that such hypersensitivity has not been confirmed, and in many cases, are not real [39–41]. To address this issue, a number of hospitals are now working on a project to de-label patients with a supposed hypersensitivity reported in their clinical history unless it is confirmed by the corresponding tests.

Another type of MRP that drew significant attention due to its severity was that of teratogenic drugs, for which 66% of the generated alerts were ignored. However, it must be considered that not all medicines act as teratogens in all trimesters of pregnancy, and PREFASEG is unable to distinguish between such cases. It is also possible that some alerts were generated for women who were no longer pregnant but who, by some registration error, maintained a pregnancy status in their health records.

Regarding the alerts related to therapeutic duplications, it was observed that approximately 70% of these alerts were ignored by clinicians. However, many such alerts were related to adjustment of the daily dose of treatment, and so it was necessary to combine presentations at different doses; this was common in the groups of antihypertensive RAS inhibitors and antidepressants, and in the replacement of amoxicillin with amoxicillin-clavulanate. In terms of the NSAIDs and paracetamol-type analgesics, it was observed that prescriptions were authorised for issuing on demand if necessary, which often generated alerts related to duplication if an attempt was made to prescribe a drug from the same pharmacological group. Another group of duplications that drew attention due to their association with a high risk of serious adverse effects that motivate hospital admissions were the oral anticoagulants [42]. During the 3-year study period, PREFASEG produced 14,903 alerts related to duplications in this group of drugs, which translated to an acceptance of 42% (6314), wherein the prescription was not continued.

In the case of the AEMPS safety alerts, an unexpected low degree of acceptance was recorded considering that these constituted specific alerts from a regulatory agency [43]. In fact, throughout the 3-year study period, the degree of acceptance of the AEMPS safety alerts decreased, and in 2018 they reflected the lowest percentage of acceptance (22%) of all alerts throughout that year. Among these notices, the Triple Whammy, which is associated with a significant increase in the risk of kidney failure [44], represented the largest number of alerts.

Upon examination of the alerts related to the use of drugs advised against in geriatrics, it was observed that the degree of acceptance ranged from 30% in 2016 to 27% in 2018. Despite the fact that this alert category is considered of low clinical relevance, wherein use of a specific drug may not be recommended in older patients but is not totally contraindicated, it produced similar or even superior acceptance results compared to the AEMPS safety alerts. It was therefore considered that this level of acceptance was due to physicians being somewhat more sensitive to safety alerts related to elderly patients. However, we must not lose sight of the fact that > 70% of these alerts were discarded and the corresponding prescriptions was generated, which could lead to potential adverse reactions in patients. In this context, it is estimated that drug-associated adverse effects produce approximately 6.5% of hospital admissions, of which more than half of these could be prevented [45–48].

The pharmacological groups that generated the highest number of alerts in geriatric patients were the benzodiazepines and the NSAIDs, which are also widely used drugs throughout the population. The significant increase in the number of alerts for benzodiazepines (i.e., from 30,662 in 2016 to 68,974 in 2018) was particularly surprising, and these were mainly attributed to alprazolam. It is known that both an advanced age, which is linked to metabolic and pharmacokinetic changes, and the number of drug treatments that a patient is receiving, are two of the situations that increase the risk of adverse drug effects to the greatest extent [49–51]. In addition, it must be considered that the world population is constantly aging, which is accompanied by a greater degree of pathologies, and an increase in the use of pharmaceuticals [52–54].

Analysing the percentages of acceptance for alerts related to the use of drugs advised against in geriatrics, significant variation was observed between the different pharmacological groups. More specifically, muscle relaxants and peripheral vasodilators were the groups with the highest degrees of acceptance. According to a previous study, physicians tend to prioritise alerts that are more clinically relevant, or that can be resolved with the least amount of time or effort [55].

In a classic study looking at hospitalisations caused by adverse effects, it was found that the majority occurred in the elderly, and were due to commonly used drugs with well-known safety profiles [56]. Considering this point, which can likely be extrapolated to other countries, it would be interesting to analyse the situation of patients for whom PREFASEG detected a possible MRP that was not addressed.

In terms of limitations to the current study, it should be noted that the moderate percentage of alert acceptance highlights the need to investigate the causes that lead clinicians to discard such a high number of recommendations. Thus, to maximise the usefulness of PREFASEG and to avoid possible alert fatigue, it will be necessary to carry out a detailed review into the traceability data of the tool to eliminate low-relevance alerts that are generated but not accepted, and to highlight any alerts related to therapeutic orientations while providing one or more alternative active ingredients. The introduction of a block to prevent the continuation of a prescription associated with a severe MRP could also be considered.

On the other hand, essential future work should also focus on analysing the acceptance of MRP alerts based on their clinical relevance and the type of recommendation, which are key aspects to consider in the case of drug interactions. In addition, a satisfaction survey should be carried out to request feedback and suggestions from practitioners with regards to improving the PREFASEG system in terms of its clinical content and technological aspects.

The future development of PREFASEG also involves the inclusion of medicines that can only be prescribed in hospitals and their corresponding contraindications, which will allow the program to be extended to different levels of care. The technological evolution of the tool is also necessary to render it more specific when generating alerts. For example, this could be achieved using the terminology common to all SNOMED CT systems (Systematised Nomenclature of Medicine—Clinical Terms) that determine the active ingredient, the dose, the pharmaceutical form, and the number of packaging units [57–59]. To optimise the use of PREFASEG and improve the management of clinical information, intelligent systems such as natural language processing could be applied that would allow the clinician to obtain and interact with the information recorded in text format in the patient's clinical history [60, 61]. An improved follow-up and monitoring of the PREFASEG alerts would also be desirable, wherein details regarding the professional receiving the alert are registered and made visible, in addition to whether this alert is ignored, and the level of care of the corresponding professional.

In summary, PREFASEG appears to be a feasible and efficient strategy to improve some aspects of clinical safety related to the prescription of drugs, and as a result, in the health care received by patients.

## Conclusions

Our study demonstrated that the PREFASEG (PREscripción FArmacéutica SEGura, i.e., safe pharmaceutical prescription) clinical decision support system contributes to the prevention of potential safety medicine-related problems in patients. In 28% of the cases in which the tool generated a safety alert, primary care physicians modified their prescriptions by some means. The main drug groups implicated in the PREFASEG alerts were the non-steroidal anti-inflammatory drugs, the benzodiazepines, and the renin-angiotensin system inhibitors; groups that frequently cause adverse effects and motivate hospital admissions.

In future, it will be necessary to study in detail the reasons behind the fact that > 70% of the generated alerts were ignored by physicians. In addition, the possibility of reducing the number of alerts should be assessed to avoid alert fatigue. Moreover, it is evident that strategies must be designed to make the prescriber aware of the importance of patient safety, as well as to technologically improve the tool and render it more robust and specific.

## Supplementary Information

**Additional file 1.** **Annex Table 1.** Description of the types of PREFASEG alerts; **Annex Table 2.** Groups of pharmacological duplications included in PREFASEG in 2018; **Annex Table 3.** Definitions of the AEMPS safety alerts included in PREFASEG in 2018; **Annex Table 4.** List of drugs not recommended for use in geriatrics in 2018.

## Data Availability

The datasets used and/or analysed during the current study are available from the corresponding author on reasonable request.

## Abbreviations

ADE: Adverse drug event
AEMPS: Spanish Agency for Medicines and Health Products (Agencia Española del Medicamentos y Productos Sanitarios)
CDSS: Clinical decision support system
ECW: Electronic clinical workstation
EHR: Electronic health record
ICS: Institut Català de la Salut (Catalan Health Institute)
MRP: Medication-related problem
NSAIDs: Non-steroidal anti-inflammatory drugs
PC: Primary care
PREFASEG: PREscripción FArmacéutica SEGura, i.e., safe pharmaceutical prescription
RAS: Renin-angiotensin system
