# Supplementary material for: Safer prescription of drugs: impact of the PREFASEG system to aid clinical decision-making in primary care in Catalonia
Source: BMC Med Inform Decis Mak. 2021 Dec 15;21:349. doi: 10.1186/s12911-021-01710-8 (PMC8675496; doi:10.1186/s12911-021-01710-8)
Supplement: Supplementary file 1 — Additional file 1. Annex Table 1. Description of the types of PREFASEG alerts; Annex Table 2. Groups of pharmacological duplications included in PREFASEG in 2018; Annex Table 3. Definitions of the AEMPS safety alerts included in PREFASEG in 2018; Annex Table 4. List of drugs not recommended for use in geriatrics in 2018. [file 12911_2021_1710_MOESM1_ESM.doc]

**SUPPORTING INFORMATION ANNEXES**

Safer prescription of drugs: Impact of the PREFASEG System to Aid Clinical Decision-making in Primary Care in Catalonia

M.Àngels Pons Mesquida^1,3^

Míriam Oms Arias^1^

Eduard Diogène Fadini^2,3^

Albert Figueras^3^

^1^Unitat de Coordinació i Estratègia del Medicament (UCEM). Institut Català de la Salut, Barcelona, Spain

^2^Servei de Farmacologia Clínica, Hospital Universitari Vall d’Hebron. Institut Català de la Salut, Barcelona, Spain

^3^Departament de Farmacologia, Terapèutica i Toxicologia. Universitat Autònoma de Barcelona

**Corresponding author email:** aponsmesquida@gencat.cat

**Annex Table 1. Description of the types of PREFASEG alerts**

| **MRP alert** | **Description** |
| --- | --- |
| **Interactions** |  |
| Contraindications for health problems | Detection of patients with absolute contraindications defined in the ICS Clinical Practice Guidelines and the CatSalut Harmonisation Guidelines for the following pathologies: Diabetes mellitus II, heart failure, chronic kidney disease, hypercholesterolemia, and depression. |
| Contraindications for clinical variables | Detection of patients with contraindications due to renal insufficiency:  • Severe renal insufficiency: glomerular filtration of 15 or   30 mL/min/1.73 m^2^  • End-stage renal failure: glomerular filtration <15   mL/min/1.73 m^2^  Detection of patients with contraindications due to abnormal potassium levels:  • Hypokalaemia <3.5 mmol/L  • Hyperkalaemia >5.5 mmol/L. |
| Contraindications with a specific AEMPS alert | Detection of patients with contraindications due to the use of an active ingredient that has a relevant safety alert from the Spanish Agency for Medicines and Health Products (AEMPS).  The active ingredients included are: Aliskiren, citalopram, escitalopram, cilostazol, trimetazidine, raloxifene and bazedoxifene, COXIBS, diclofenac, aceclofenac, ivabradine, agomelatine, canagliflozin, and the “Triple Whammy” (i.e., NSAIDs + diuretics + RAS inhibitors). |
| Teratogenic pharmaceuticals | Detection of female patients with contraindications based on the use of active ingredients in pregnancy (diagnoses registered in the ECW). Teratogenic drugs are considered those who fall into categories D and X of the old FDA Reproductive Risk Classification. It should be noted that in June 2015, the FDA decided to remove this classification. |
| Drugs advised against for use in geriatrics | Detection of patients >75 years of age who are taking any inappropriate medication that exhibits a more unfavourable risk-benefit profile due to their age. |
| Duplicate therapies | Detection of patients who are classed as receiving a non-beneficial prescription of two or more drugs with the same active ingredient (alone or in combination) and/or with the same pharmacological action.  Duplicities considered relevant and duplicities considered dose adjustments (i.e., combinations sought at the therapeutic level) are clearly differentiated. |
| Anticholinergic drug combinations | Detection of patients receiving a prescription of any urinary antispasmodic together with another drug that exhibits significant anticholinergic effects (e.g., antihistamines, tricyclic antidepressants, etc.). |
| History of suspected hypersensitivity | Detection of patients for whom a history of unconfirmed hypersensitivity has been recorded. |
| Adverse drug events (ADEs) | Detection of patients for whom an ADE has been registered. |

**ANNEX Table 2. Groups of pharmacological duplications included in PREFASEG in 2018**

| **TYPE OF DRUG OR SYSTEM UPON WHICH THE DRUG ACTS** | **TYPE OF DUPLICATION** |
| --- | --- |
| CARDIOVASCULAR SYSTEM | 1. Antithrombotic agents: acetylsalicylic acid (Aspirin) 2. Antiplatelet agents: clopidogrel 3. Oral anticoagulants 4. Digitalis 5. Antiarrhythmics |
| ANTIHYPERTENSIVES | 1. Diuretics: thiazides 2. Loop diuretics 3. Potassium sparing diuretics 4. β-Blockers 5. Non-dihydropyridine calcium antagonists 6. Dihydropyridine calcium antagonists 7. High blood pressure medications: renin-angiotensin system inhibitors (ACEIs, AIIRAs) 8. α-Adrenergic antagonists |
| ANTIULCER AGENTS | 1. Gastric protectors 2. Antacids |
| MUSCULOSKELETAL SYSTEM | 1. Anti-inflammatories 2. Anti-gout agents: allopurinol 3. Anti-gout agents: uricosuric 4. Anti-gout agents: colchicines 5. Analgesics: metamizole 6. Analgesics: paracetamol 7. Minor opiates 8. Triptans |
| ANTIBIOTICS | 1. Penicillins 2. Tetracyclines 3. Macrolides 4. Quinolones |
| HYPOLIPIDEMIC AGENTS | 1. Statins 2. Other lipid-modifying agents 3. Fibrates |
| ANXIOLYTICS AND HYPNOTICS | 1. Long-acting benzodiazepines 2. Intermediate-acting benzodiazepines 3. Short-acting benzodiazepines 4. Z-Drugs and others |
| ANTIEPILEPTICS | 1. Barbiturates 2. Other antiepileptics |
| ANTIDEPRESSANTS | 1. Tricyclic antidepressants 2. SSRI antidepressants 3. Other antidepressants (I) 4. Other antidepressants (II) 5. Bupropion and naltrexone |
| ORAL ANTIDIABETICS | 1. Biguanides 2. Secretagogues (sulfonylureas and glinides) 3. Glitazones 4. Incretins (glyptins and GLP-1 analogues) 5. Type 2 sodium-glucose cotransporter inhibitors (ISGLT-2) 6. Long-acting insulin (injected form) |
| GENITOURINARY SYSTEM | 1. Urinary antispasmodics 2. Prostate drugs: testosterone inhibitors |
| SYSTEMIC HORMONAL PREPARATIONS | 1. Systemic corticosteroids 2. Aromatase inhibitors 3. Osteoporosis treatments 4. Oestrogens and progestogens |
| RESPIRATORY SYSTEM | 1. Long-acting β2-agonists 2. Anticholinergics 3. Inhaled glucocorticoids |
| OTHERS | 1. Calcium supplements 2. Potassium supplements 3. Iron supplements 4. Magnesium supplements 5. Vitamin D |

**ANNEX Table 3. Definitions of the AEMPS safety alerts included in PREFASEG in 2018**

| **DRUG** | **ATC** | **YEAR OF ALERT PUBLICATION** | **ALERT CRITERIA** |
| --- | --- | --- | --- |
| Citalopram | N06AB04 | 2011 | High doses:  - Above 40 mg/day.  - Above 20 mg/day in patients >65 years of age.  - Above 20 mg/day in patients suffering from liver dysfunction.  Administered in combination with other drugs that also prolong the QT interval of the electrocardiogram. |
| Escitalopram | N06AB10 | 2011 | High doses (>10 mg/day in patients >65 years of age).  Administered in combination with other drugs that also prolong the QT interval of the electrocardiogram. |
| Aliskiren | Aliskiren alone (C09XA02)  Aliskiren + hydrochlorothiazide (C09XA52)  Aliskiren + amlodipine (C09XA53)  Aliskiren + hydrochlorothiazide + amlodipine (C09XA54) | 2014 | In patients with a diagnosis of diabetes mellitus II or undergoing treatment with antidiabetic drugs (ATC A10).  Jointly administered with ACE inhibitors (ATC: C09AA, C09BA, C09BB) and/or ARA-IIs (ATC: C09CA, C09DA, C09DB, C09DX). |
| Cilostazol | B01AC23 | 2013 | In patients suffering from a health problem where its use is contraindicated, i.e., cerebral haemorrhage, severe ventricular arrhythmias, or heart failure.  Or, in concomitant treatment with:  - 2 Antiplatelet agents  - Antiplatelet + oral anticoagulant |
| Trimetazidine | C01EB15 | 2012 | In patients with a diagnosis of extrapyramidal and movement disorders. |
| Raloxifene or bazedoxifene | Raloxifene (G03XC01)  Bazedoxifene (G03XC02) |  | In patients suffering from any health problem where it is contraindicated, e.g., venous thromboembolism, uterine sac, endometrial cancer, or liver failure of any degree. |
| COXIBS | Celecoxib (M01AH01)  Etoricoxib (M01AH05) |  | In patients suffering from any health problem where it is contraindicated, e.g., ischemic heart disease, peripheral arterial disease, cerebrovascular disease, heart failure, or inflammatory bowel disease. |
| Diclofenac or Aceclofenac | Diclofenac alone (M01AB05) or combined (M01AB55)  Aceclofenac (M01AB16) | 2013, 2014 | In patients suffering from any health problem where its use is contraindicated, e.g., ischemic heart disease, peripheral arterial disease, cerebrovascular disease, or heart failure. |
| Agomelatine | N06AX22 | 2014 | In patients ≥75 years of age. |
| Ivabradine | N06AX22 | 2014 | Co-administration with verapamil (C08DA01, C08DA51, C09BB10) or (C08DB01). |
| “Triple Whammy” (NSAIDs + RAS inhibitors + diuretics) | NSAIDs alone and in combination | 2014 | In patients ≥75 years of age or undergoing treatment for diabetes (ATC A10). |
| Canagliflozin | A10BK02  Metformin + canagliflozin (A10BD16) | 2016, 2017 | In patients suffering from a health problem in which it is necessary to be more careful due to an increased risk of amputation. |

**ANNEX Table 4. List of drugs not recommended for use in geriatrics in 2018**

| **A03A: AGENTS AGAINST FUNCTIONAL ALTERATIONS OF THE STOMACH** | | **M03B: CENTRAL ACTION MUSCLE RELAXANTS** | |
| --- | --- | --- | --- |
| A03AB06 | Otilonium bromide | M03BA03 | Methocarbamol |
| **A03F: PROKINETIC AGENTS** | | M03BX02 | Tizanidine |
| A03FA01 | Metoclopramide | M03BX07 | Tetrazepam |
| **A10B: HYPOGLYCEMANTS (EXCEPT INSULINS)** | | M03BX08 | Cyclobenzaprine |
| A10BB01 | Glibenclamide | **N02A: OPIOIDS** | |
| A10BB02 | Clorpropamide | N02AB02 | Pethidine |
| **B01A: ANTITHROMBOTICS** | | N02AD01 | Pentazocine |
| B01AC05 | Ticlopidine | **N02C: ANTIMIGRANE AGENTS** | |
| B01AC07 | Dipyridamole | N02CA01 | Dihydroergotamine |
| B01AC22 | Prasugrel | N02CA51 | Combinations with  Dihydroergotamine |
| B01AC24 | Ticagrelor | N02CA52 | Combinations with ergotamine |
| B01AC23 | Cilostazol | **N04A: ANTIPARKINSONIAN AGENTS** | |
| **C02A:  CENTRAL ACTION ANTIADRENERGICS** | | N04AA01 | Trihexyphenidyl |
| C02AB01 | Methyldopa | **N05B: ANSIOLYTICS and N05C: HYPNOTICS AND SEDANTS** | |
| C02AC01 | Clonidine | **SHORT- AND INTERMEDIATE-ACTING BENZODIAZEPINES** | |
| C02AC05 | Moxonidine | N05BA08 | Bromazepam (INTERMEDIATE) |
| **C02C: PERIPHERAL ACTION ANTIADRENERGICS** | | N05BA12 | Alprazolam |
| C02CA01 | Prazosine | N05BA14 | Pinazepam (INTERMEDIATE) |
| C02CA04 | Doxazosin | N05BA21 | Clotiazepam |
| G04CA03 | Terazosin | N05BA91 | Bentazepam |
| **C04A: PERIPHERAL VASODILATORS** | | N05CD05 | Triazolam |
| C04AD03 | Pentoxifylline | N05CD08 | Midazolam |
| C04AE02 | Nicergoline | N05CD09 | Brotizolam |
| C04AX21 | Naftidrofuryl | N05CD11 | Loprazolam |
| **C07A:  BETA-BLOCKING AGENTS** | | **LONG-ACTING BENZODIAZEPINES** | |
| C07AA07 | Sotalol | N03AE01 | Clonazepam (N03A) |
| **G04B: OTHER UROLOGICAL PRODUCTS, INCLUDING ANTI-SPASMODIC PRODUCTS** | | N05BA02 | Clordiazepoxid |
| G04BD04 | Oxybutynin | N05BA05 | Clorazepat dipotassium salt |
| **L02A: HORMONES AND RELATED AGENTS** | | N05BA09 | Clobazam |
| L02AB01 | Megestrol | N05BA10 | Ketazolam |
| **M01A: NSAIDs: NON-STEROIDAL ANTI-INFLAMMATORY AND ANTI-RHEUMATIC** | | N05BA13 | Halazepam |
| M01AA01 | Phenylbutazone | N05BA51 | Combinations with diazepam |
| M01AB01 | Indomethacin | N05BA55 | Combinations with clorazepat dipotassium salt |
| M01AB15 | Ketorolac (H) | N05CD01 | Flurazepam |
| M01AB16 | Aceclofenac | N05CD03 | Flunitrazepam |
| M01AB05 | Diclofenac | N05CD10 | Quazepam |
| M01AB55 | Combinations with diclofenac | **NON-BENZODIAZEPINE HYPNOTICS** | |
| M01AC01 | Piroxicam | N05CF02 | Zolpidem |
| M01AC02 | Tenoxicam | N05CF01 | Zopiclone |
| M01AC05 | Lornoxicam | **R06A: ANTIHISTAMINES FOR SYSTEMIC USE** | |
| M01AC06 | Meloxicam | **H1 ANTIHISTAMINES 1ST GENERATION** | |
| M01AE03 | Ketoprofen | R06AB06 | Dexbrompheniramine |
| M01AE09 | Flurbiprofen | R06AB56 | Combinations with dexbrompheniramine |
| M01AE14 | Dexibuprofen | R06AB01 | Brompheniramine |
| M01AE17 | Dexketoprofen | R06AB51 | Combinations with brompheniramine |
| M01AG01 | Mefenamic acid | R06AX07 | Triprolidine |
| M01AH01 | Celecoxib | R06AA02 | Dimenhydrinate |
| M01AH05 | Etoricoxib | R06AA09 | Doxylamine |
| M01AX01 | Nabumetone | R06AA52 | Combinations with diphenhydramine |
| **N06A: ANTIDEPRESSANTS** | | R06AB02 | Dexchlorpheniramine |
| **TRICYCLIC ANTIDEPRESSANTS** | | R06AC01 | Mepyramine |
| N06AA12 | Doxepine >6mg/d | R06AD01 | Alimemazine |
| N06AA02 | Imipramine | R06AD02 | Promethazine |
| N06AA04 | Clomipramine | R06AD03 | Tiethylperazine |
| N06AA06 | Trimipramine | R06AD07 | Mequitazine |
| N06AA21 | Maprotiline | R06AE05 | Meclozine |
| **SSRIs** | | R06AE92 | Combinations with clocinizine |
| N06AB03 | Fluoxetine | R06AX02 | Cyproheptadine |
| **N06B:PSYCHOSTIMULANTS AND NOOTROPICS** | | R06AX17 | Ketotifen |
| N06BX03 | Piracetam | R06AX19 | Azelastine |
| **R03D: OTHER SYSTEMIC AGENTS AGAINST OBSTRUCTION OF THE RESPIRATORY TRACT** | | H02BX92 | Clemastine |
| R03DA04 | Theophylline | N05BB01 | Hydroxyzine |
